# Surgery after combination therapy with a tyrosine kinase inhibitor and anti-PD-1 antibody in sarcomatoid hepatocellular carcinoma: case report and literature review

**DOI:** 10.3389/fonc.2023.1212013

**Published:** 2023-10-05

**Authors:** Bin Liang, Tao Huang, Shao-Lei Kuang, Guang-Yuan Xie, Tian-Qi Liu, Yuan-Yuan Chen

**Affiliations:** ^1^ Department of Hepatobiliary, Pancreatic and Spleen Surgery, Guangxi Academy of Medical Sciences, the People’s Hospital of Guangxi Zhuang Autonomous Region, Nanning, China; ^2^ Department of General Surgery, the Jiangbin Hospital of Guangxi Zhuang Autonomous Region, Nanning, China

**Keywords:** sarcomatoid hepatocellular carcinoma, anti-angiogenic therapy, anti-PD-1 antibody, surgery, combination therapy

## Abstract

**Introduction:**

Although surgery is the preferred treatment for sarcomatoid hepatocellular carcinoma (SHC), the prognosis remains considerably poor due to early postoperative recurrence and metastasis. Reports on surgery after combined treatment with a tyrosine kinase inhibitor and anti-programmed cell death (PD)-1 antibody are unavailable.

**Case presentation:**

A 69-year-old male patient with SHC was admitted to our hospital for treatment of a liver tumor that was detected on ultrasonography. Abdominal computed tomography with triple-phase enhancement revealed a lesion in the right hepatic lobe that measured 86.0 mm × 75.0 mm × 71.0 mm. Biopsy revealed a pathological diagnosis of liver sarcoma or sarcomatoid carcinoma. The patient subsequently received transcatheter arterial chemo-embolization, as he did not consent to surgery. More than two months later, he received a combination of lenvatinib with camrelizumab, as computed tomography showed an increase in the lesion size (to 123.0 mm × 90.0 mm × 80.0 mm) and lateral growth posterior to the upper pole of the right kidney. Liver resection was performed after 6 months of systemic therapy; pathological examination confirmed a diagnosis of SHC and showed extensive necrosis of tumor cells. Combined treatment with lenvatinib and camrelizumab was continued for 6 months after surgery. The patient has survived for over 24 months after initial diagnosis and is currently tumor-free.

**Conclusion:**

Combined systemic therapy with a tyrosine kinase inhibitor and anti-PD-1 antibody may represent a feasible treatment strategy for improving resectability in cases of unresectable SHC. The outcomes with this combination may also be explored in cases of resectable SHC that have a high-risk of recurrence; this may improve the therapeutic effect.

## 1 Introduction

Sarcomatoid hepatocellular carcinoma (SHC) is a rare subtype of primary liver cancer, which accounts for approximately 0.09-1.40% of all hepatocellular carcinomas (1–5). The pathogenesis and underlying mechanisms involved in the development of SHC remain unclear. However, it is known that it demonstrates a higher degree of malignancy than conventional hepatocellular carcinoma and is associated with more rapid growth, earlier recurrence, and a poorer prognosis despite radical resection (4). Surgery is currently the preferred treatment for SHC, as the efficacy of alternative treatments such as radiotherapy, chemotherapy, and targeted therapy remain uncertain (6). However, owing to the absence of any obvious symptoms in the early stage, most cases are beyond the scope of surgery at diagnosis. Following the breakthrough with combined treatment (using a tyrosine kinase inhibitor [TKI] and anti-programmed cell death [PD]-1 antibody) for hepatocellular carcinoma, researchers have explored the feasibility of using the combination in SHC. In this context, a recent report suggested that a patient with advanced SHC demonstrated complete response following immunotherapy (7). Combining surgical resection with systemic treatment may therefore represent an effective approach for improving the chances of cure in SHC. Studies are therefore needed to evaluate the feasibility of using systemic therapy for improving resectability in unresectable cases. This report describes a case of unresectable SHC where surgical resection was possible after combined treatment with a TKI and anti-PD-1 antibody; the combined approach showed excellent therapeutic effect. It also provides a comprehensive overview of the treatment of SHC.

## 2 Case presentation

**Figure 1:**
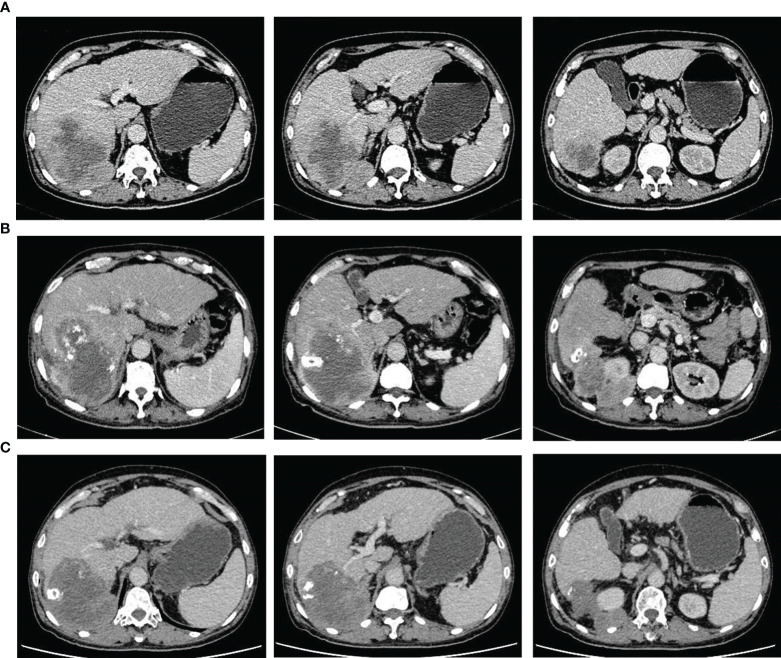
Representative computed tomography images showing changes in the tumor throughout the treatment period prior to surgery. **(A)** perihepatic tumor with blurred edges in the right liver at initial diagnosis. **(B)** extrahepatic tumor growth (with a blurred boundary) covering the upper pole of the right kidney, 2 months after transcatheter arterial chemoembolization. **(C)** stable tumor size and clear boundary after treatment with tyrosine kinase inhibitors and anti-PD-1 antibodies for 6 months.

A 69-year-old male patient was admitted to the Department of Gastroenterology after being diagnosed with liver cancer during routine physical examination on March 20, 2021. He had suffered from chronic hepatitis B (HB) infection 23 years previously, and had received antiviral therapy for 1 year; however, the course and outcomes of the treatment were unclear. He reported no history of other underlying diseases or any significant family history. No remarkable positive signs were detected on physical examination (including abdominal tenderness and pain on percussion of the liver, among others). Laboratory findings showed an albumin concentration of 34.2 g/L; the levels of HB surface, e, and core antibodies were 155.89 mIU/ml, 4.20 PEI U/ml, and 7.60 PEI U/ml, respectively, and those of HB viral deoxyribonucleic acid were < 500 IU/mL. Among the tumor markers, the levels of protein induced by vitamin K absence or antagonist-II were slightly increased (165 mAU/mL), while those of alpha-fetoprotein, carcinoembryonic antigen, carbohydrate antigen 125, and carbohydrate antigen 19-9 were within the normal range. Enhanced computed tomography (CT) revealed the presence of liver cirrhosis with a tumor in the right lobe of the liver that measured 86.0 mm × 75.0 mm × 71.0 mm and was considered to be malignant (**Figure 1A**). No other abnormalities were found on examination and he did not complain of any aberrant symptoms. The patient had Child-Pugh class A liver function and an Eastern Cooperative Oncology Group performance status score of 0.

### 2.1 Diagnosis and treatment

**Figure 2:**
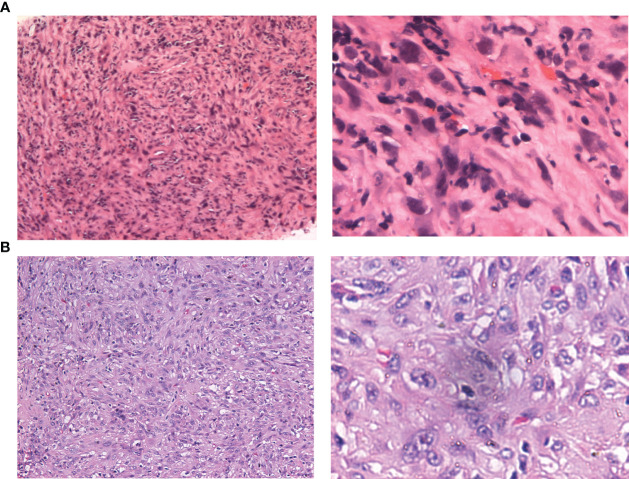
Hematoxylin-eosin staining of needle puncture biopsy and resected specimens. **(A)** original magnification of 100× and 400× in puncture biopsy specimen. **(B)** original magnification of 100× and 400× in resected specimen.

The patient was transferred to the Department of Intervention Therapy, as he did not consent to surgery. Ultrasound-guided needle biopsy of the tumor demonstrated a pathological diagnosis of hepatic malignancy in favor of a sarcoma or sarcomatoid carcinoma (**Figure 2A**). Transcatheter arterial chemoembolization (TACE) was performed under local anesthesia on March 30, following which the patient gradually developed dull pain on the right side of the waist and abdomen. A review of laboratory data two months later showed the albumin level to be low (at 23.3 g/L); the levels of protein induced by vitamin K absence or antagonist-II had reverted to normal (20 mAU/mL). However, enhanced CT showed enlargement of the tumor (in the right lobe of the liver), which now measured 123.0 mm × 90.0 mm × 89.0 mm (**Figure 1B**). In view of the poor outcome with interventional therapy, the patient was referred to our department for evaluation.

**Figure 3:**
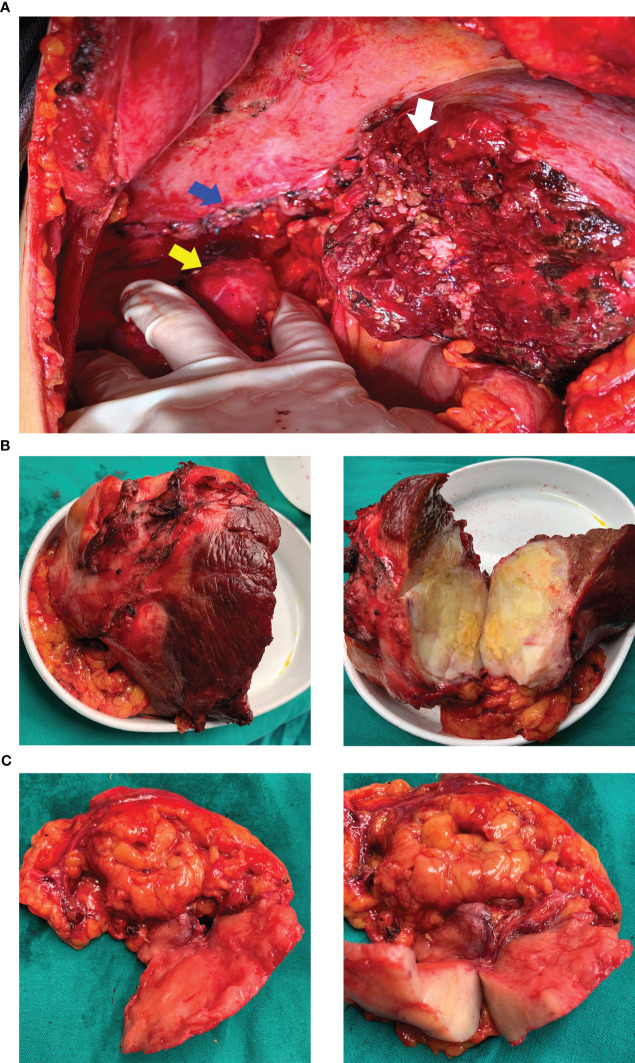
Surgical field and resected specimen. **(A)** surgical field after resection of the tumor. The white arrow shows the section of the right liver, the blue arrow indicates the sutured and repaired right diaphragm, and the yellow arrow shows the naked upper pole of the right kidney after resection of the perirenal fat sac. **(B)** resected specimen of the right liver. **(C)** resected specimen of the right diaphragm and tissues around the upper pole of the right kidney.

**Figure 4:**
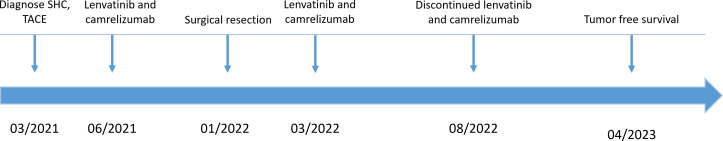
Timeline of treatment course.

After multi-disciplinary discussion, he was diagnosed with an unresectable hepatic malignancy; radical surgical resection was considered to be impossible due to indistinct intrahepatic tumor boundaries, severe liver cirrhosis, and the possibility of malignant invasion of the right diaphragm and kidney. The available therapeutic options included radiotherapy, chemotherapy, antiangiogenic therapy, and immune checkpoint inhibitors. After consideration of the available options, he finally received lenvatinib (at a dose of 8 mg once a day) in combination with intravenous camrelizumab (200 mg every 3 weeks). The dull pain started reducing after three months and adverse effects including gingival bleeding, hoarseness, joint pain, and cramps also began to appear gradually; however, they were mild and tolerable and required no specific treatment. After treatment with a TKI and anti-PD-1 antibody for 6 months, enhanced CT showed a reduction in the size of the liver tumor, which now measured 110.0 mm × 90.0 mm × 76.0 mm. In addition, the boundary between the tumor and the surrounding tissue, both inside and outside the liver, had become more distinct (**Figure 1C**). Based on the modified Response Evaluation Criteria in Solid Tumors, the tumor response was evaluated as stable disease; the patient was therefore scheduled for tumor resection on January 7, 2022 after discontinuation of lenvatinib for approximately 15 days. Moderate to severe liver sclerosis was observed intraoperatively and the tumor was found to be located behind the posterior lobe of the liver (right side); the boundary between the tumor and diaphragm was indistinct and it encompassed the upper pole of the right kidney. The liver tumor was removed completely in addition to part of the right diaphragm and right perirenal fat sac that were attached to the tumor (**Figure 3**). The timeline of the course of treatment is shown in **Figure 4**.

### 2.2 Outcomes and follow-up

**Figure 5:**
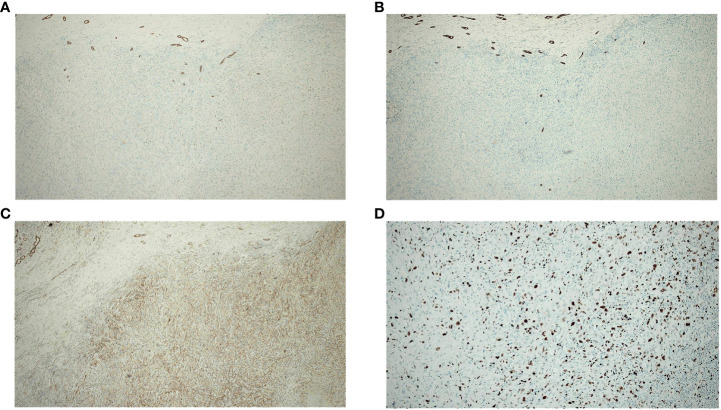
Immunohistochemistry of spindle cells in the resected specimen (**A–C**) show positivity for CK, CK19, and β-catenin. **(D)** shows Ki-67 positivity in approximately 40% of cells.

On gross pathological examination of the resected specimen, the lesion in the right liver measured 102 mm × 85 mm × 67 mm; the tissue from the right side of the diaphragm and that around the upper pole of the right kidney measured 77 mm × 36 mm × 20 mm; the sections of the tumor appeared grayish white, solid, and tough. Microscopically, the specimens showed a neoplasm in the liver tissue and alien spindle and epithelioid cells within the mass. A diffuse, patchy, invasive growth was seen that had destroyed the surrounding liver tissue; examination also showed vacuolated nuclei, visible eosinophilic nucleoli, interstitial hyaline changes, necrosis of tumor cells, rare instances of nuclear fission, and small foci of hyperplasia in the bile ducts (**Figure 2B**). On immunohistochemistry, the spindle cells tested positive for cytokeratin (CK), CK19, integrase interactor 1, leukocyte common antigen, and β-catenin and Ki-67 positivity was observed in approximately 40% of cells (**Figure 5**); however, they tested negative for cluster of differentiation (CD) 30, caudal type homeobox-2, carcinoembryonic antigen, CK7, CK20, isocitrate dehydrogenase-1, and tumor protein 53. The CD68 (+) cells that were found in the interstitium tested negative for mouse double minute 2 on fluorescence *in situ* hybridization. The tumor was finally diagnosed as SHC based on pathological examination. The patient was discharged after 12 days of surgery, without any postoperative complications. Combined treatment with lenvatinib and camrelizumab was restarted at 1 month after surgery and discontinued after maintenance therapy for 6 months. During follow-up, the patient underwent biochemical tests for evaluation of liver function and alpha-fetoprotein and protein induced by vitamin K absence or antagonist-II levels; he also underwent ultrasonography and CT every 3–6 months. During the latest follow-up visit on April 10, 2023, his general condition was found to be good and there were no signs of recurrence.

## 3 Discussion

SHC is a rare histological subtype of primary liver cancer, that is characterized by the proliferation of spindle and pleomorphic cells with or without multinucleated giant cells (2). Although its pathogenesis remains unclear, previous reports have shown that SHC may respond to various anticancer therapies including TACE, radiofrequency ablation, and percutaneous ethanol injection (8–12). In this context, certain reports have suggested that infections with hepatitis B/C viruses may contribute to sarcomatoid transformation (6, 13–15). However, an increasing number of reports on SHC indicate no history of treatment with anticancer or antiviral agents (16, 17).

Similar to patients described in most reports, our patient did not receive any antineoplastic therapy at the time of diagnosis; however, it is unclear whether SHC may have developed following previous antiviral therapy for HB infection. Notably, although our patient did not receive TACE before diagnosis, the tumor progressed continuously after he underwent TACE. The tumor increased in size from 8.6 cm to 14.0 cm (mainly outside the liver) within 2 months; the extrahepatic infiltration may be attributed to the fact that the tumor needed to obtain a supply of blood after the supplying hepatic artery was embolized. This suggests that TACE may not be effective and may even promote tumor growth in such cases; it therefore needs to be used with caution in SHC, especially in peripheral tumors.

Similar to hepatocellular carcinoma, SHC is asymptomatic in the early stages and most are advanced by the time symptoms appear. Epigastric discomfort is the most common symptom during the first hospital visit; this may be related to the rapid enlargement of the tumor and invasion of the liver capsule, peritoneum, and diaphragm (13). Physical examination in our case revealed an intrahepatic tumor with a maximum diameter of 8 cm; however, there were no symptoms. Upper abdominal pain appeared gradually with enlargement of the tumor; the patient also demonstrated typical clinical manifestations. As there are no specific tumor markers for SHC, imaging is the mainstay for detection of the lesions (18).

On the CT/magnetic resonance imaging scan, SHC usually presents as a large mass with an uneven density/signal, peripheral enhancement, central necrosis, variable enhancement of the solid portion with or without the tumor capsule, and intrahepatic metastasis (17, 19, 20). The CT scan in our case showed the mass to be located in the posterior lobe of the liver (right side). The lower edge of the mass protruded from the liver capsule, and demonstrated peripheral and progressive enhancement; no enhancement was observed in the central area. After six months of combined targeted and immunotherapy, the tumor boundaries appeared more distinct on CT imaging; although there was an insignificant change in the tumor volume, this indicated that the treatment was effective. The diagnosis of SHC mainly relies on pathological examination. Dual phase components of cancer and sarcoma can be seen under the light microscope, with extensive dedifferentiation and spindle cell morphology. On immunohistochemistry, the tumor tests positive for epithelial and mesenchymal markers; CK and vimentin are also expressed and the Ki-67 index is high (21, 22).

SHC has a poor prognosis and is more aggressive than hepatocellular carcinoma (5, 23). The 1, 3, and 5-year overall survival rates of patients with SHC have been reported to be 20.4%, 8.0%, and 5.7%, respectively. In a study, the overall survival rates for SHC were found to be 29.3%, 17.4%, and 8.11% at 6, 12, and 18 months, respectively, regardless of the treatment received (24). Several studies indicate radical surgical resection to be the most effective treatment for SHC (15, 24). A retrospective study that included 63 cases of resected SHC showed the median overall and recurrence-free survival duration to be 23.2 and 8.4 months, respectively; the 1-, 2-, and 3-year overall survival rates were 59.0%, 45.4%, and 34.4%, respectively, and the 1-, 2-, and 3-year recurrence-free survival rates were 36.3%, 26.1%, and 22.4%, respectively (25). Morisue et al. (5) reported the estimated 1-, 3- and 5-year overall survival rates of resectable SHC to be 64.3%, 32.1%, and 16.1%, respectively. However, some studies have reported a poorer prognosis for SHC, with a median overall survival of 5.8 months despite surgery. They also reported the cumulative survival at 6 and 12 months to be 44.0% and 4.0%, respectively; recurrence (both intrahepatic and extrahepatic) was observed in all patients (26).

Recurrence and metastasis are the limiting factors that affect the efficacy of surgery in SHC; this may be related to the clinical characteristics of the tumor. Studies have shown that patients with SHC demonstrate a larger tumor size; lower incidence of tumor encapsulation; higher rates of tumor necrosis, adjacent organ invasion, and lymph node metastasis; and more advanced differentiation grade and TNM stage than conventional hepatocellular carcinoma (3, 13, 27). Liver transplantation is an option for patients with early stage SHC, who achieve a similar prognosis as those undergoing liver resection; however, chemotherapy and radiotherapy offer unsatisfactory therapeutic outcomes in both palliative and adjuvant settings (1, 24, 28). In recent years, there have been few reports of TACE and radiofrequency ablation treating SHC, as previous reports suggested that may be inducing factors for SHC (26). The hypovascular nature of SHC may be responsible for the poor survival benefit offered by TACE (29).

Recent trials using treatments based on molecular targeted therapy and immunotherapy have demonstrated improvements in survival; this has expanded the treatment options for advanced hepatocellular carcinoma (30, 31). Studies have shown that the TKI, apatinib, can prevent disease progression in pulmonary sarcomatoid carcinoma; it exhibits high efficacy and prolongs overall survival in cases treated with immune checkpoint inhibitor therapy (32, 33). Researchers have therefore explored the use of TKIs and immune checkpoint inhibitors in SHC. Reports suggest that SHC demonstrates high rates of mutation in *CDKN2A*, *EPHA5*, *FANCM*, *MAP3K1*, *TP53*, *TERT*, and *KRAS*; *CDKN2A* mutation, *NTRK1* fusion, and *BRCA1/2* mutation may represent potential therapeutic targets in patients with SHC (3, 34). In addition, approximately 33.0–40.0% of SHCs express PD-ligand-1, which is associated with prognosis and may be a potential biomarker for immunotherapeutic treatment (5, 35). In their report, Goto et al. (36) described a case of SHC in which the tumor demonstrated attenuation of arterial enhancement within 1 month of administration of lenvatinib; however, discontinuation of the drug owing to adverse effects led to tumor regrowth. Subsequent combined treatment with atezolizumab and bevacizumab led to tumor shrinkage, which was maintained for 3-8 months. In their report, Zhu et al. (7) described a case of SHC that had relapsed after surgery and progressed after successive administration of sorafenib and ranvartinib. However, complete response was achieved after subsequent use of nivolumab; this indicated considerable reduction of the metastatic lesions to undetectable sizes. Fencer et al. (37) reported a case of unresectable SHC, where the patient achieved disease control for over a year after receiving atezolizumab and bevacizumab; this warrants further investigation of this combination (of antiangiogenic therapy and immunotherapy). Although numerous studies have reported various potential therapeutic targets and cases have been effectively treated with TKI and anti-PD-1 antibodies in the clinic, a clear basis for recommending the use of these regimens (or guiding drug selection) is currently lacking.

A few studies have reported on the treatment of SHC using antiangiogenic and immune checkpoint inhibitor therapy; all existing reports pertain to palliative treatments for unresectable or recurrent SHC. In our case, tumor progression was first observed after TACE; subsequent treatment using a combination of a TKI and anti-PD-1 antibodies achieved disease control and surgical resection was successfully performed. The patient has survived for over 24 months after initial diagnosis and is currently tumor-free.

This is the first report on surgical resection of SHC after combined treatment using a TKI and anti-PD-1 antibody. Notably, the intrahepatic lesion was removed first and the extrahepatic part was removed subsequently due to difficulties in exposure during surgery; this confers high risk for early postoperative recurrence. The satisfactory outcomes in this case may be attributed to the use of lenvatinib and camrelizumab before and after surgery; combining a TKI with an anti-PD-1 antibody may therefore represent an optimal strategy for improving chances of resection in unresectable SHC. The findings suggested that a change in the characteristics of the tumor boundary (from indistinct to clear on imaging) may indicate suitability for surgical resection. Although the findings in this case are promising, a single case may not represent the entire population of patients with SHC. Further clinical studies are needed to facilitate the widespread use of this new treatment approach.

## 4 Conclusion

In conclusion, SHC is a highly invasive malignant tumor which demonstrates a poor prognosis despite surgery (which is the preferred treatment). TKIs and anti-PD-1 antibodies have shown benefits in certain cases; their use in unresectable SHC, and subsequent surgery after appropriate evaluation, may improve patient outcomes and lead to its use as a recommended treatment strategy. However, the approaches for screening suitable patients need to be explored. In addition, the postoperative administration of TKIs and anti-PD-1 antibodies in cases with high-risk of recurrence warrant further investigation.

## Data availability statement

The raw data supporting the conclusions of this article will be made available by the authors, without undue reservation.

## Ethics statement

The studies involving humans were approved by the Medical Ethics Committee of the People’s Hospital of Guangxi Zhuang Autonomous Region. The studies were conducted in accordance with the local legislation and institutional requirements. Written informed consent for participation was not required from the participants or the participants' legal guardians/next of kin in accordance with the national legislation and institutional requirements. Written informed consent was obtained from the individual(s) for the publication of any potentially identifiable images or data included in this article. Written informed consent was obtained from the participant/patient(s) for the publication of this case report.

## Author contributions

BL collected all references and wrote the draft. TH, S-LK, and G-YX helped collect case data and explore writing ideas. T-QL and Y-YC conceived and designed the study. Y-YC revised the manuscript, and discussed the meaning of the manuscript. All authors contributed to the article and approved the submitted version.
